# Patterns of Change in Athletic Identity After Anterior Cruciate Ligament Reconstruction

**DOI:** 10.3390/ijerph22010076

**Published:** 2025-01-08

**Authors:** Britton W. Brewer, Rachel Shinnick, Allen E. Cornelius, Judy L. Van Raalte, Fahimeh Badiei

**Affiliations:** 1 Department of Psychology, Springfield College, 263 Alden Street, Springfield, MA 01109, USA; jvanraal@springfieldcollege.edu (J.L.V.R.); f.badiei77@gmail.com (F.B.); 2 Department of Counseling & School Psychology, University of Massachusetts Boston, 100 Morrissey Boulevard, Boston, MA 02125, USA; rachelshinnick@gmail.com; 3 School of Psychology, Fielding Graduate University, 2020 De La Vina Street, Santa Barbara, CA 93105, USA; allencornelius@gmail.com

**Keywords:** knee, self-identity, surgery

## Abstract

Changes in athletic identity have been documented after injury and other sport transitions in nomothetic investigations. Patterns of change in athletic identity after injury have not been examined systematically at the individual level. In the current study, secondary analyses were performed on two data sets (*N* = 43 and *N* = 80) in which athletic identity values were available for before and at least six months after anterior cruciate ligament (ACL) reconstruction. A stable pattern of athletic identity was most common (48–68% of participants), followed, respectively, by a decreasing pattern (19–45% of participants) and an increasing pattern (7–14% of participants) in both data sets, with a trend toward a decreasing pattern over time in the data set in which athletic identity values were available up to two years after surgery. Partial support was obtained for the claim that decreases in athletic identity after ACL surgery are related to postoperative perceptions of knee symptoms and function. The current intraindividual findings complement the results of nomothetic studies and suggest that although stability of athletic identity after sport injury seems to be the norm, changes in athletic identity are also common and should be considered in applied work with athletes who have sustained injuries.

## 1. Introduction

Sport injury can be a substantial source of stress [1] and, consistent with the integrated model of psychological response to sport injury [2], can affect the cognition, emotion, and behavior of athletes [3]. As proposed by Wiese-Bjornstal et al. [2], self-perceptions are among the cognitive factors that can be influenced by sport injury. In support of this contention, changes in both self-esteem [4] and self-identity [5,6,7] have been documented after sport injury. Given that sport can be a prominent source of self-worth and self-identification for athletes [8] and that injury can threaten sport involvement, it is understandable that post-injury alterations in self-related cognition may occur.

Self-identity, which refers to how people define themselves in terms of the goals, values, and beliefs that they find personally expressive and to which they are firmly committed [9], has been examined extensively in the context of sport injury literature in the form of “athletic identity” [10]. In accordance with evidence that people’s conceptions of themselves are both stable and malleable [11], athletic identity has been viewed as displaying consistency over time while remaining susceptible to contextual influences [12]. For athletes, injury may serve as a contextual influence on self-identity. In support of this argument, reductions in athletic identity were documented between 6 and 12 months after anterior cruciate ligament (ACL) reconstruction, with greater decreases reported by individuals with slower recovery progress [5]. The analyses on which these findings were based, however, reflect the aggregated responses of participants in the study sample, potentially obscuring the full range of responses by individual participants. Thus, although the sample as a whole reported a statistically significant decrease in athletic identity during the postoperative period in question, individual participants may have exhibited a wider array of responses.

### 1.1. Patterns of Change in Athletic Identity

For any given period of time in which athletic identity is assessed at a starting point and an ending point, there are three possible patterns of change: (a) decrease in athletic identity (i.e., weaker identification with the athlete role); (b) stable athletic identity (i.e., no change); and (c) increase in athletic identity (i.e., stronger identification with the athlete role). Theoretical explanations for each of the possible patterns can be provided in response to the occurrence of a serious sport injury. A decrease in athletic identity after injury could represent a form of self-protection in which athletes seek to maintain a positive self-image in the face of a threat to an important domain of self-identity by psychologically distancing themselves from that domain [13]. Thus, by devaluing athletic identity, athletes make the threat to sport involvement posed by injury less relevant to self-evaluation and self-definition [14]. Decreases in athletic identity have been documented after not only sport injury [5], but after other potentially psychologically threatening events as well, such as a poor competitive season [15], sport team deselection [16], and sport career termination [17,18,19]. Some athletes have decreased their identification with the athlete role proactively, before disengaging from competitive sport involvement, to forestall subsequent identity-related difficulties [20,21].

The rationale for expecting athletes to maintain a stable athletic identity after sustaining a serious sport injury lies in the trait-like properties of self-identity (i.e., consistency over time). Stability in athletic identity could offer athletes a degree of inner cohesion in their sense of self during the potentially turbulent times that sport injury can bring. In accordance with continuity theory [22], athletes may view transitional situations such as injury through the lens of their previous experiences of themselves and their social environment, using coping strategies that enable them to preserve and maintain their internal psychological characteristics and social relationships.

A potential explanation for an increase in athletic identity after sport injury can be found in an investigation by Benson et al. [23] in which priming athletes with thoughts about an end to their sport career elicited higher athletic identity scores than a control condition (i.e., priming thoughts about dental pain). The goal-discrepant cognitions associated with sport career termination presumably prompted the athletes to “double down” on their athletic identity, a phenomenon that has been documented in response to a serious sport injury [24]. Athletic identity promotion of this sort would be consistent with the general process model of psychological threat [25], which holds that the uncertainty of a looming or unresolvable threat prompts defensive responses that involve increasing commitment to personally or socially important domains.

### 1.2. The Current Study

Although changes in athletic identity have been documented for groups (and subgroups) after sport injury [5] and other threats to sport involvement [15,16,18,19] in nomothetic investigations, individual trajectories have largely been neglected in quantitative research. Consequently, the purpose of the current study was to examine individual patterns of change in athletic identity after ACL surgery and rehabilitation through secondary analysis of previously published data [5] and athletic identity data collected as part of a second prospective longitudinal study of ACL surgery and rehabilitation [26]. Using the method of identifying patterns of change after a threatening event developed by Bonanno et al. [27], individual trajectories in athletic identity were examined from just prior to ACL reconstruction to 6 months after ACL reconstruction in two independent samples. Given the argument of Benson et al. [23] that “although a more exclusive focus on athletic identity may reduce the anxious uncertainty induced by goal-discrepant thoughts in the short-term, this defensively motivated response may ultimately be a long-term maladaptive issue for individuals” (p. 312), it is possible that individuals may tend toward adopting an adaptive course of action and, in so doing, be less likely to increase their identification with the athlete role with the passage of time after ACL surgery. Consequently, a secondary purpose of the current study was to examine the extent to which patterns of change in athletic identity after ACL surgery vary over time through analysis of data collected from one sample just prior to ACL reconstruction and 6, 12, and 24 months after ACL reconstruction. Similarly, given that an inverse relationship has been documented between decrements in athletic identity after ACL surgery and recovery progress [5], a third purpose of the current study was to investigate associations between patterns of change in athletic identity following ACL reconstruction and the self-reported impact of knee-related symptoms on their sport activity level and ability to engage in various sport activities.

## 2. Materials and Methods

### 2.1. Participants

Data were collected from two samples of participants. Sample A consisted of 43 patients (23 women and 20 men) with a mean age of 34.09 (*SD* = 12.24) years who served as participants in a randomized trial examining the effects of an interactive cognitive-behavioral multimedia program on rehabilitation outcomes [25]. Participants in Sample A self-identified as predominantly White (93%), not Hispanic or Latino (93%), and involved in sport as competitive (*n* = 18, 42%) or recreational (*n* = 21, 49%) athletes. Sample B consisted of 80 patients (27 women and 53 men) with a mean age of 29.01 (*SD* = 10.07) years who served as participants in a prospective longitudinal study of psychological responses to ACL surgery and rehabilitation [5]. Participants in Sample B self-identified as predominantly White (90%), not Hispanic or Latino (94%), and involved in sport as competitive (*n* = 39, 49%) or recreational (*n* = 39, 49%) athletes. Demographic characteristics of participants in Sample A and Sample B are presented in Table 1.

### 2.2. Measures

Demographic and injury-related variables, athletic identity, and subjective rehabilitation outcome variables were measured for both Sample A and Sample B. Demographic and injury-related information (e.g., age, gender, race/ethnicity, date of ACL injury, level of sport involvement) was obtained through a survey. The Athletic Identity Measurement Scale (AIMS) [28], a 7-item survey with responses given on a scale from 1 (strongly disagree) to 7 (strongly agree), was used to measure athletic identity. The AIMS, for which an alpha coefficient of 0.81 has been obtained [28], has been widely implemented to assess identification with the athlete role [29]. As documented in a meta-analysis [29], AIMS scores are moderately associated with measures of sport-related intrinsic motivation and commitment to sport (*r* = 0.51). Of particular relevance to the current study, the AIMS has been used to detect changes in athletic identity after exposure to events that threaten sport involvement [5,6,15,16,18,19]. The Knee Outcomes Survey—Sports Activities Scale (KOS-SAS) [30], a 10-item questionnaire with responses given on a scale from 0 to 5, was used to assess respondents’ perceptions of the impact of knee-related symptoms on their sport activity level and ability to engage in various sport activities. Individual item scores are summed and doubled to create a scale with a maximum total score of 100 such that high scores correspond with fewer knee-related symptoms and higher levels of sport functioning. KOS-SAS scores are strongly correlated with scores on similar measures of knee symptoms and functioning [30]. Internal consistency was acceptable (α = 0.91) in both Sample A and Sample B.

### 2.3. Procedure

**Figure 1:**
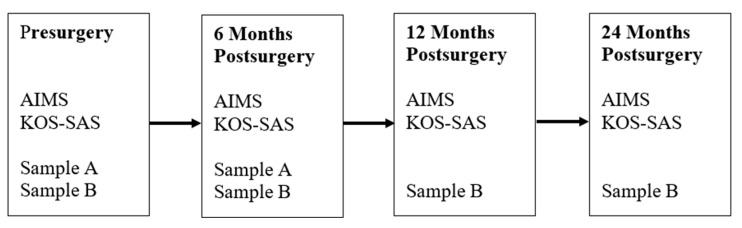
Changes in individual participants’ AIMS scores for decreasing, increasing, and stable patterns of change in athletic identity.

The study was conducted in accordance with the Declaration of Helsinki. Springfield College Institutional Review Board (IRB) approval was obtained prior to data collection for both Sample A and Sample B. Prior to experiencing ACL reconstruction with one of the collaborating surgeons, participants in both samples completed an informed consent document and a series of measures that included a survey requesting demographic and injury-related information, the AIMS, and the KOS-SAS. Participants in Sample A completed the AIMS and KOS-SAS again at approximately 6 months postsurgery, and participants in Sample B completed the AIMS and KOS-SAS again at approximately 6, 12, and 24 months postsurgery. An overview of the study design is presented in Figure 1.

As part of the randomized controlled trial in which they were enrolled, participants in Sample A were randomly assigned to either an experimental condition or a control condition after the preoperative assessment. Whereas participants in the control condition received standard care, participants in the experimental condition received an interactive cognitive-behavioral multimedia program designed to increase knowledge about ACL reconstructive surgery, decrease psychological distress before ACL reconstructive surgery, reduce pain and reinjury anxiety after ACL reconstructive surgery, and improve outcomes after ACL reconstructive surgery. No information pertaining to self-identity was provided to any of the participants in the study. Participants in Sample A received compensation of USD 150 for full participation in the study. Participants in Sample B received USD 60 for providing data prior to surgery and at approximately 6, 12, and 24 months after surgery.

### 2.4. Data Analysis

Consistent with methods used by Bonanno et al. [27] to examine changes in depression following bereavement and adapted by Shapiro et al. [31] to study patterns of emotional response after ACL reconstruction, changes in athletic identity were determined by comparing participants’ postsurgical AIMS scores (6 months for Sample A and 6, 12, and 24 months for Sample B) with their presurgical AIMS scores. Participants whose postsurgical AIMS scores exceeded their presurgical AIMS scores by a magnitude greater than the pooled standard deviation for the sample’s (postsurgical AIMS score—presurgical AIMS score) difference score were considered to have displayed an increase in athletic identity. Participants whose presurgical AIMS scores exceeded their postsurgical AIMS scores by a magnitude greater than the pooled standard deviation for the sample’s (postsurgical AIMS score—presurgical AIMS score) difference score were considered to have displayed a decrease in athletic identity. Participants whose postsurgical AIMS scores differed from their presurgical AIMS scores by a magnitude less than the pooled standard deviation for the sample’s (postsurgical AIMS score—presurgical AIMS score) difference score were considered to have displayed a pattern of stable athletic identity.

To determine whether group assignment had an effect on the variables examined for Sample A, a series of independent sample *t*-tests comparing the experimental and control conditions was performed on presurgical and postsurgical AIMS and KOS-SAS scores. A series of one-way analyses of variance (ANOVAs) was performed to compare the KOS-SAS scores of participants across the increasing, decreasing, and stable patterns of change in athletic identity for Sample A and for each of the three postoperative assessment episodes for Sample B. Partial eta-squared and Cohen’s *d* effect sizes were calculated for the group comparison analyses where appropriate.

## 3. Results

Means and standard deviations of presurgical and postsurgical AIMS and KOS-SAS scores for Samples A and B are presented in Table 2. Results pertaining to patterns of change in athletic identity are presented separately for Sample A and Sample B.

### 3.1. Sample A

None of the independent sample *t*-tests comparing the experimental and control conditions that were performed on presurgical and postsurgical AIMS and KOS-SAS scores were statistically significant. Therefore, data from participants in the experimental condition were considered together with those from participants in the control condition in subsequent analyses, and group assignment was not used as a covariate.

**Figure 2:**
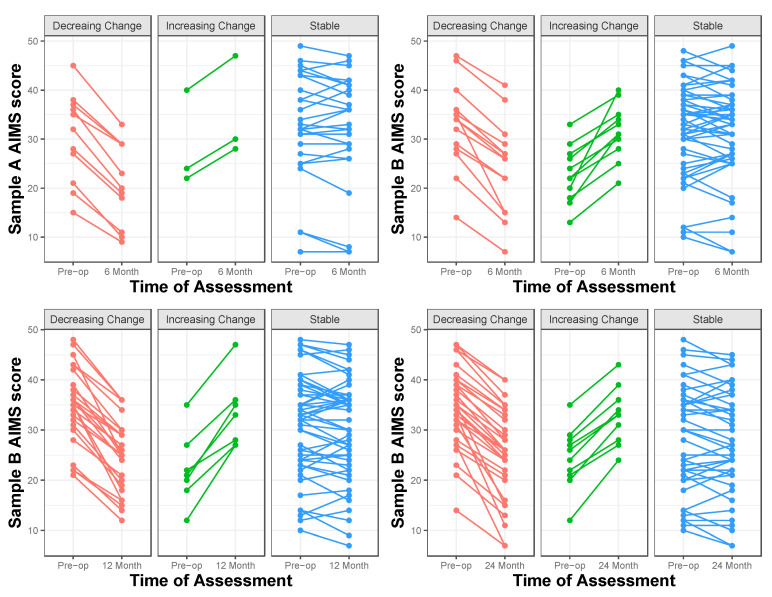
Changes in individual participants’ AIMS scores for decreasing, increasing, and stable patterns of change in athletic identity over 6 months (for Samples A and B), 12 months (for Sample B), and 24 months (for Sample B).

**Figure 3:**
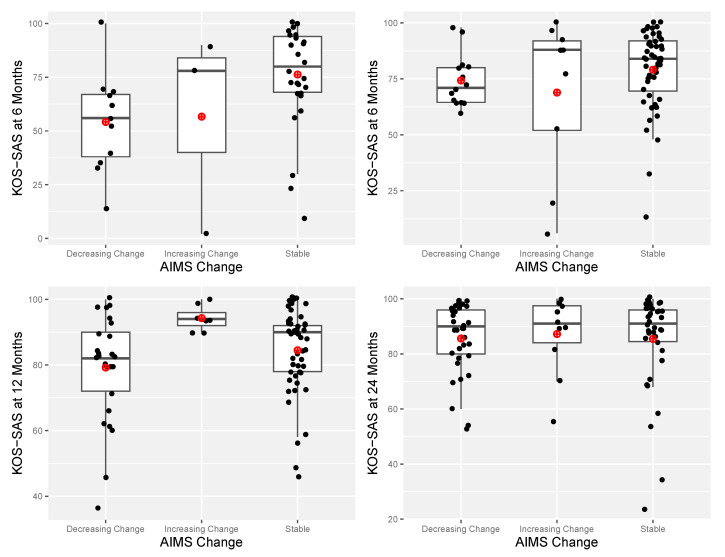
Differences in KOS-SAS scores for three groups (decreasing change, increasing change, and stable) for Samples A and B. Black dots correspond to scores of individual participants and red dots correspond to group mean scores.

The mean AIMS change score from before surgery to six months after surgery was −2.44 (*SD* = 5.19). Consequently, participants whose AIMS scores increased by more than 5 were considered to have shown an increasing pattern of athletic identity, and participants whose AIMS scores increased by 5 or less or decreased by 5 or less were considered to have shown a stable pattern of athletic identity, and participants whose AIMS scores decreased by more than 5 were considered to have shown a decreasing pattern of athletic identity. As shown in Table 3 and the upper panel of Figure 2, a few participants demonstrated an increasing pattern of change in athletic identity, the majority of participants demonstrated a stable pattern, and a substantial minority of participants demonstrated a decreasing pattern. In the one-way ANOVA comparing the KOS-SAS scores of participants across the increasing, decreasing, and stable patterns of change in athletic identity, a significant effect of the pattern was obtained, *F*(2,39) = 3.39, *p* = 0.04, η^2^ = 0.15. Postsurgical KOS-SAS scores for participants with a stable pattern of athletic identity (*M* = 76.29, *SD* = 23.64) were significantly higher (*p* = 0.02) than those for participants with a decreasing pattern of athletic identity (*M* = 54.18, *SD* = 23.21, Cohen’s *d* = 0.94) and not significantly different (*p* = 0.21) from those for participants with the increasing pattern of athletic identity (*M* = 56.67, *SD* = 47.72, Cohen’s *d* = 0.52) (see upper left panel in Figure 3).

### 3.2. Sample B

The mean AIMS change scores from before surgery to 6 months, 12 months, and 24 months after surgery were −0.86 (*SD* = 5.92), −2.75 (*SD* = 6.77), and −3.15 (*SD* = 6.67), respectively. Consequently, at all three postoperative time points, participants whose AIMS scores increased by 6 or more were considered to have shown an increasing pattern of athletic identity, participants whose AIMS scores increased by 5 or less or decreased by 5 or less were considered to have shown a stable pattern of athletic identity, and participants whose AIMS scores decreased by 6 or more were considered to have shown a decreasing pattern of athletic identity. As shown in Table 3 and Figure 3, the percentage of participants demonstrating an increasing pattern was low across all three time points, the percentage of participants demonstrating a stable pattern diminished steadily across the three time points, and the percentage of participants demonstrating a decreasing pattern increased steadily across the three time points. In the one-way ANOVAs comparing the KOS-SAS scores of participants across the increasing, decreasing, and stable patterns of change in athletic identity, no significant effects of the pattern were obtained in the 6-month, *F*(2,72) = 1.22, *p* = 0.30, η^2^ = 0.03, 12-month, *F*(2,64) = 2.84, *p* = 0.07, η^2^ = 0.08, and 24-month, *F*(2,64) = 0.23, *p* = 0.80, η^2^ = 0.01, postoperative assessments. There was, however, a significant difference in postoperative KOS-SAS scores across the patterns of change groupings for the 12-month assessment, *F*(2,78) = 3.51, *p* = 0.03, η^2^ = 0.08. Postoperative KOS-SAS scores for participants with the increasing pattern (*M* = 94.28, *SD* = 3.73) were significantly higher (*p* = 0.01) than those for participants with the decreasing pattern (*M* = 79.20, *SD* = 16.23, Cohen’s *d* = 1.28). This difference is likely due to the unusual clustering of high KOS-SAS scores for the increasing pattern group.

## 4. Discussion

The primary aim of the current study was to examine individual patterns of change in athletic identity after ACL reconstruction, with a secondary purpose of assessing the extent to which patterns of change in athletic identity after ACL reconstruction vary over time. To accomplish these objectives, a secondary analysis was performed on two data sets in which longitudinal postoperative athletic identity data were collected. For one of the data sets, athletic identity values were obtained prior to surgery and at 6, 12, and 24 months after surgery. Across two independent samples, the predominant pattern of athletic identity was that of stability, ranging from 48% to 68% across assessment points. For many participants, consistency in athletic identity may have offered a sense of continuity in oneself while experiencing the challenges of ACL surgery, rehabilitation, and return to activity. Despite the prominence of the stable athletic identity pattern, however, the finding that 30–52% of participants demonstrated a change either up or down in athletic identity at any given time point after ACL surgery corroborates results of qualitative studies documenting turbulence in self-identity after the occurrence of sport injury [24,32,33].

Few participants in either of the two samples (i.e., 7–14%) demonstrated a pattern of increasing athletic identity in any of the assessments. Increasing athletic identity may be, as Benson et al. [23] noted, possible or desirable only for a limited period of time. Although some individuals may diminish the threat to athletic identity posed by ACL surgery or may derive (or attempt to derive) motivational benefit during ACL rehabilitation through the expansion of athletic identity, the viability and effectiveness of such an approach may only be temporary for most individuals given the immediate and sustained reduction in physical capacity after ACL reconstruction.

In contrast, the pattern of decreasing athletic identity seemed to grow more prevalent with the passage of time after ACL surgery, progressing from 19 to 26% at 6 months postsurgery to 45% at 24 months postsurgery. This trend mirrors the one documented in the nomothetic analysis of Sample B data [5]. The reason for the trend is not clear. Although it has been suggested that the trend may reflect a self-protective mechanism in response to potentially diminished sport success [5], other possible explanations include participants reprioritizing their commitments after experiencing postsurgical limitations or simply following a developmental trend of decreased athletic identity with age as intensity of sport involvement wanes [8].

A third purpose of the study was to investigate associations between patterns of change in athletic identity following ACL reconstruction and the self-reported impact of knee-related symptoms on their sport activity level and ability to engage in various sport activities. Although only significant at 6 months for Sample A and 12 months for Sample B, the association between patterns of change in athletic identity and self-reported knee symptoms and function reflected a large effect size at 6 months for Sample A, a medium effect size for 12 months for Sample B, and a small effect size for 6 months and 24 months for Sample B. These effect size findings suggest that changes in athletic identity after ACL surgery are tied to recovery status only for a limited period of time, after which they may be related more strongly to other factors (e.g., success in returning to sport). Further longitudinal research with additional predictor variables is needed to verify this interpretation of the current results.

The main strengths of the current investigation are the use of a prospective longitudinal research design, the inclusion of long-term follow-up collection of data, and the replication of analyses across multiple samples of participants. Limitations of the study include the use of a questionnaire to assess changes in athletic identity and, relatedly, exclusive reliance on self-report data. Although the measure of athletic identity is well-validated and has been widely used in athletic identity research [34], quantitative assessment could be augmented with qualitative methods to inquire how, why, and to what end(s) their self-identity did or did not change. Similarly, in future research, self-reports could be corroborated through the incorporation of other indicators of changes in self-identity (e.g., other reports, self-drawings [35], and self-representations on social media). It is also important to note that the method for determining what constituted a change in athletic identity in the current study was based on that developed by Bonanno et al. [27]. Adopting other criteria for labeling meaningful intraindividual changes in athletic identity might have revealed a different pattern of findings. It is, however, noteworthy that the results for Sample B are largely consistent with those reported by Brewer et al. [5] for the same participants despite major differences in statistical methods.

A critical issue to examine in future investigations is the extent to which patterns of change (or stability) after ACL surgery are adaptive. It is possible that depending on the athlete, a given change in athletic identity may be either maladaptive or adaptive. For example, a decrease in athletic identity for some athletes may reflect a “loss of identity” (i.e., a subtraction of a portion of self-identity that is not replaced by a comparable increase in some other aspect of identity) and be accompanied by distress [36,37], whereas for other athletes, such a decrease in athletic identity may be replaced by a corresponding increase in other domains of self-identity that facilitate acceptance and adaptation. Similarly, an increase in athletic identity may boost motivation during rehabilitation but may also create unrealistic expectations and delay or prolong difficulties in adjustment. Even maintaining the same level of athletic identity during rehabilitation may be beneficial (e.g., by facilitating continuity of the self across challenging circumstances) or harmful (e.g., if the current/future functional reality is widely discrepant with the maintained level of athletic identity).

As noted by Nyland and Pyle [38], it is possible that lower levels of athletic identity could help reduce the risk of post-injury psychological difficulties. Psychoeducational interventions with the potential to reduce levels of athletic identity are available [39,40], but these interventions were designed for use with athletes for transitions out of competitive sport, namely deselection [40] and completion of intercollegiate sport eligibility [39]. It is unclear how implementing such interventions after the occurrence of sport injury would be received by athletes and whether they would have a salubrious effect on psychological adjustment.

Although the current study was narrowly focused on a single facet of self-identity (i.e., athletic identity) and a single threat to self-identity (i.e., ACL surgery and rehabilitation), the analytical approach and findings may have broader implications for research outside the realm of sport. Both theory [41] and research [42] support considering the impact of identity on behavior in a wide array of domains (e.g., social, volunteer, health, consumer, and entrepreneurial). Changes in identity may have far-reaching ramifications for behavior in those and are, therefore, worthy of investigation in future research.

## 5. Conclusions

The general tendency was for athletic identity to remain stable for up to two years after ACL reconstruction. A substantial portion of individuals, however, reported marked increases and, more typically, decreases in athletic identity following ACL surgery. Postoperative changes in athletic identity corresponded at least partially to recovery status, consistent with the position that levels of athletic identity may both influence and be influenced by events and experiences surrounding ACL surgery and rehabilitation.

## Figures and Tables

**Table 1:** Demographic characteristics of participants in Sample A and Sample B.

Variable	Sample A	Sample B
Gender	23 women, 20 men	27 women, 53 men
Age (years)	*M* = 34.09 (*SD* = 12.24)	*M* = 29.01 (*SD* = 10.07)
Sport involvement ^1^	42% C, 49% R, 9% N	49% C, 49% R, 2% N

^1^ C = competitive athlete; R = recreational athlete; N = nonathlete.

**Table 2:** Means (and standard deviations) for AIMS and KOS-SAS scores for Samples A and B.

Sample	Presurgery	6 Months	12 Months	24 Months
Sample A				
AIMS	31.00 (10.69)	28.56 (11.79)		
KOS-SAS	28.05 (23.53)	69.10 (26.78)		
Sample B				
AIMS	31.20 (9.47)	30.34 (9.32)	28.61 (9.12)	27.63 (9.49)
KOS-SAS	42.05 (24.62)	76.99 (19.54)	83.67 (14.13)	86.60 (14.16)

Note. AIMS = Athletic Identity Measurement Scale; KOS-SAS = Knee Outcomes Survey—Sports Activities Scale. Score ranges are 7–49 for the AIMS and 0–100 for the KOS-SAS.

**Table 3:** Frequencies (and percentages) of patterns of change in athletic identity after ACL reconstruction for Samples A and B.

Sample	Increasing Pattern	Stable Pattern	Decreasing Pattern
Sample A	3 (7%)	29 (67%)	11 (26%)
Sample B			
6 months	11 (14%)	54 (68%)	15 (19%)
12 months	7 (8%)	29 (67%)	26 (31%)
24 months	5 (7%)	34 (48%)	32 (45%)

Note. For Sample A, increasing pattern = AIMS scores increased by >5 and decreasing pattern = AIMS scores decreased by >5. For Sample B, increasing pattern = AIMS scores increased by >6 and decreasing pattern = AIMS scores decreased by >6.

## Data Availability

The data presented in this study are available on request from the corresponding author.
